# Using Graphene Sulfonate Nanosheets to Improve the Properties of Siliceous Sacrificial Materials: An Experimental and Molecular Dynamics Study

**DOI:** 10.3390/ma13214824

**Published:** 2020-10-28

**Authors:** Hongyan Chu, Zifei Wang, Yu Zhang, Fengjuan Wang, Siyi Ju, Lanxin Wang, Danqian Wang

**Affiliations:** 1 College of Civil Engineering, Nanjing Forestry University, Nanjing 210037, China; 2 Jiangsu Key Laboratory of Construction Materials, School of Materials Science and Engineering, Southeast University, Nanjing 211189, China; wangzifei97@126.com (Z.W.); tgyuzhang@outlook.com (Y.Z.); jusiyi1997@163.com (S.J.); wanglanxinvanessa@foxmail.com (L.W.); 3 Advanced and Innovative Materials (AIM) Group, Department of Civil, Environmental and Geomatic Engineering, University College London, London WC1E 6BT, UK; danqian.wang.16@ucl.ac.uk

**Keywords:** mechanical properties, graphene sulfonate nanosheets (GSNs), pore structure, ablation behavior, sacrificial materials

## Abstract

The fabrication of high-performance cement-based materials has benefited greatly from the extensive use of graphene and its derivatives. This paper studies the effects of graphene sulfonate nanosheets (GSNSs) on sacrificial cement paste and mortar (the tested materials) and other siliceous sacrificial materials, especially their ablation behaviors and mechanical properties. Decomposition temperatures and differential scanning calorimetry were used to examine how different contents of GSNSs determines the corresponding decomposition enthalpy of the tested materials and their ablation behaviors. Molecular dynamics was also used to clarify the mechanism how the GSNSs work in the CSH (calcium silicate hydrated)/GSNSs composite to increase the resistance to high temperature. The experimental results show that: (1) the contents of GSNSs at 0.03 wt.%, 0.1 wt.%, and 0.3 wt.% brought an increase of 10.97%, 22.21%, and 17.56%, respectively, in the flexural strength of siliceous sacrificial mortar, and an increase of 1.92%, 9.16%, and 6.70% in its compressive strength; (2) the porosity of siliceous sacrificial mortar was decreased by 5.04%, 9.91%, and 7.13%, respectively, and the threshold pore diameter of siliceous sacrificial mortar was decreased by 13.06%, 35.39%, and 24.02%, when the contents of GSNSs were 0.03 wt.%, 0.1 wt.%, and 0.3 wt.%, respectively; (3) a decline of 11.16%, 28.50%, and 61.01% was found in the ablation velocity of siliceous sacrificial mortar, when the contents of GSNSs were 0.03 wt.%, 0.1 wt.%, and 0.3 wt.%, respectively; (4) when considering the ablation velocities and mechanical properties of siliceous sacrificial materials, 0.1 wt.% GSNSs was considered to be the optimal amount; (5) the GSNSs contribute to the reinforced effect of GSNSs on CSH gel through the grab of dissociated calcium and water molecules, and the chemical reaction with silicate tetrahedron to produce S–O–Si bonds. These results are expected to promoting the development of new kinds of siliceous sacrificial materials that contain GSNSs.

## 1. Introduction

Siliceous sacrificial materials, due to its encasing function, are widely used to prevent radioactive materials from leaking in serious nuclear accidents. In the European Pressurized Water Reactor, sacrificial material is an important part of core catcher. Sacrificial materials can lead to lower corium temperature when melting and mixing with corium (a mixture of various structural materials, non-volatile fission products, partially or totally oxidized cladding and fuel material). In addition, radioactive fission products can be enwrapped by the matrix formed by molten SiO_2_ and Zr in the corium can be oxidized by the SiO_2_ from sacrificial concrete [1]. Thermal properties of siliceous sacrificial materials, especially their ablation behavior, contribute greatly to the mitigation of serious nuclear accidents. Since siliceous materials are increasingly applied in nuclear power plants worldwide, it is particularly significant to investigate siliceous sacrificial materials and their ablation behavior.

Currently, findings and advancement in the field of nanotechnology bring more possibilities for the improvement in the properties of cementitious composites via nanomaterials, including nano-silica [2,3,4,5], nano-titanium oxide [6], carbon nanotubes [7,8,9,10], and nano-graphite [11]. A great deal of attention has been paid to the new carbon nanomaterial, known as graphene in the section of science and engineering. In particular, graphene is featured with its 2D structure [12], as well as preeminent thermal, mechanical, electrical and optical properties [13,14]. Consequently, graphene is expected to be widely applied in cementitious composites. Nevertheless, it has some main drawbacks, including that it is not easy to disperse, and is expensive to produce. In order to cut down the production of graphene, graphene oxide nanosheets [15,16,17,18,19,20,21], graphene nanosheets and other derivatives of graphene have been invented. By side of graphene oxide sheets, there are oxygen functional groups which can improve the dispersion property of graphene oxide nanosheets [22].

Recently, the relation between graphene or its derivatives and cement-based materials has attracted the attention of a great number of researchers. Hou et al. [23] reported that the incorporation of 0.16% graphene oxide into the cement matrix resulted in an increase of 11.62% in the flexural strength of the cement matrix. An increase of 216% was found by Ranjbar et al. [24] in the flexural strength of geopolymer, when adding 1 wt.% graphene nanosheets. Lv et al. [25] found an increase of 78.6% in the tensile strength of cementitious composite, when 0.03 wt.% graphene oxide nanosheets were added. Graphene oxide nanosheets can form ordered microstructure through regulating cement hydration products [26]. As a consequence, cement-based materials can get improvement in their mechanical properties because of the addition of graphene oxide nanosheets. In addition, cementitious materials can obtain certain self-sensing capability with the help of graphene oxide nanosheets and graphene nanosheets [19], similarly to carbon fiber [27]. As demonstrated by Long et al., [28], the flexural strength of GO-mortar increased by 22% to 41.3%, when 0.20 wt.% graphene oxide was added. According to relevant studies, the mechanical properties of cement-based materials can be improved significantly by incorporating a small amount of graphene or its derivatives, but their improved effects vary dramatically in different literatures and the optimal amount of graphene or its derivatives is still controversial.

As a type of new derivative of graphene, graphene sulfonate nanosheets (GSNSs) are a kind of novel nanomaterial that is useful for improving the properties of cement-based materials. In particular, the production of GSNSs requires a lower cost than the abovementioned derivatives of graphene [29], which makes them have enormous potential in the civil engineering field. Tang et al. have made an experimental study regarding the GSNSs on the effects of precipitated calcium hydroxide morphology [30], and on the hydration of tricalcium silicate [31], which laid a foundation for the utilization of GSNSs in cement-based materials. Despite the great number of researches on graphene or its derivatives, less attention has been paid to the relation between GSNSs and ablation and mechanical properties of cement-based materials, particularly siliceous sacrificial materials.

At present, the detailed process of the interactions between cement-based materials and graphene or its derivatives is still unclear, due to the restrictions of experimental instruments and methods [30]. However, Tang et al. [30] and Hou et al. [23] have used molecular dynamics to study the interfacial bonding and strengthening mechanism of graphene or its derivatives reinforced cement-based materials, and their work proves that it is feasible to investigate the detailed interfacial connections and interactions between cement-based materials and graphene or its derivatives via molecular dynamics. In such a scenario, molecular dynamics was performed to probe the mechanism how graphene derivatives work in the cement-based materials to increase the resistance to high temperature.

The primary objective of the experiment is to explore the change in ablation behavior and mechanical properties of the tested materials with different contents of GSNSs. To this end, a comprehensive study was conducted to analyse the relationship between different contents of GSNSs and the thermal properties, pore structure, compressive strength and flexural strength of the tested materials. In addition, in accordance with the results of decomposition temperatures and differential scanning calorimetry, the decomposition enthalpy of the tested materials was identified. The corresponding ablation behavior was also included in the analysis. Simultaneously, molecular dynamics was carried out to clarify the mechanism how the GSNSs work in the CSH/GSNSs composite to increase the resistance to high temperature.

## 2. Experiment

### 2.1. Materials

**Figure 1:**
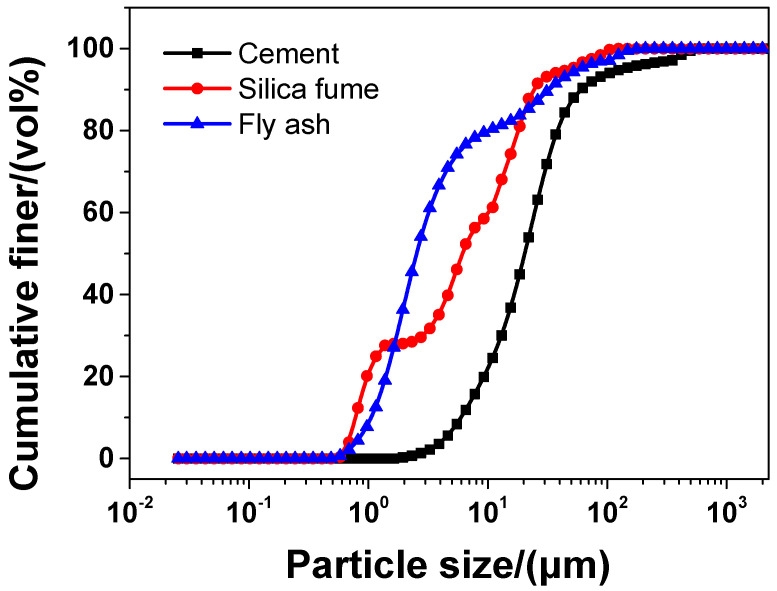
The particle size distribution of cement, silica fume, and fly ash.

**Figure 2:**
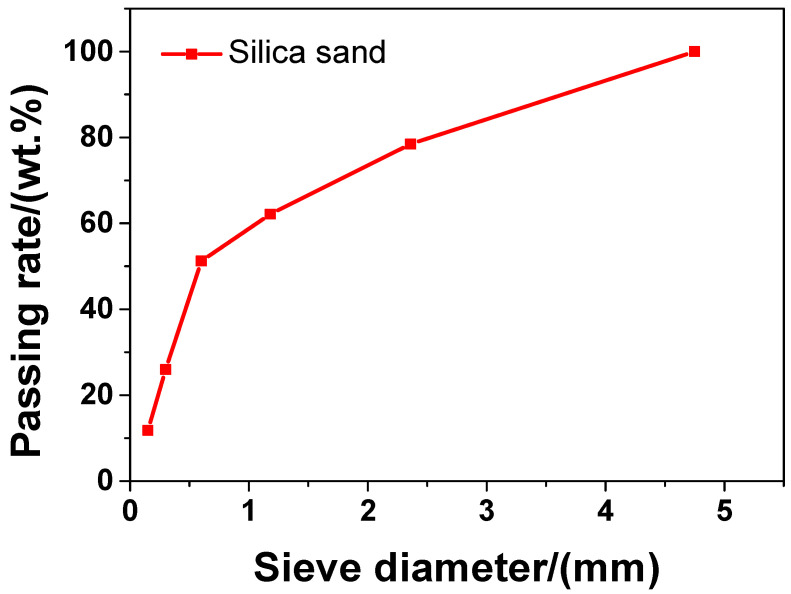
The grading curve of the silica sand.

**Figure 3:**
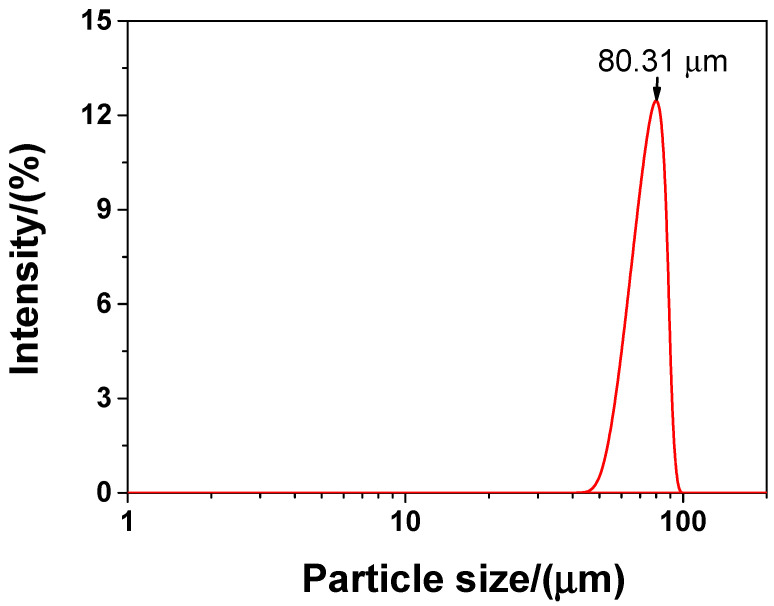
The actual particle size of the GSNSs suspension via dynamic light scattering test.

In the experiment, the main material was P·II 52.5 Portland cement (Chinese standard GB 175-2007 [32]) supplied by Jiangnan-Xiaoyetian Cement Co., Ltd. (Nanjing, China). The supplementary cementitious materials included ClassI (Chinese standard GB/T 1596-2005 [33]) fly ash produced by Zhuhai-Minghui Trading Co., Ltd. (Zhuhai, China) and silica fume produced by Aiken International Trading (Shanghai) Co., Ltd. (Shanghai, China). Table 1 shows the physical properties and chemical composition of all the materials used in the experiment, which was measured by the authors. The particle size distribution of cement, silica fume, and fly ash is presented in Figure 1 Silica sand used was obtained from a company in Liuzhou, China, known as Nuclear Industry Nonmetallic Mineral Powders Co., Ltd. SiO_2_, CaCO_3_, and MgCO_3_ were the chemical compositions of silica, accosting for 99.85%, 0.076%, and 0.074%, respectively in terms of weight fraction. The grading curve of silica sand is shown in Figure 2. A company in Nanjing, China, known as Sobute New Materials Co., Ltd., provided the superplasticizer of polycarboxylate, which was applied to achieve an ideal fluidity of sacrificial paste and mortar. As for the superplasticizer, its water-reducing rate and solid content were 33.9% and 40.0 wt.%, respectively. A company in Suzhou, China, known as Graphene-Tech Co., Ltd., was the producer and supplier of GSNSs suspension. GSNSs had a particle size of 50–100 μm and a thickness of 1–2 nm; the solid content of GSNSs was 10.5 wt.%. The actual particle size distribution of the GSNSs suspension by utilizing dynamic light scattering tests (UK Malvern Instruments Co. Ltd., Malvern, UK) is shown in Figure 3. It can be seen from Figure 3 that the GSNSs were disturbed in the range of 51.24–94.83 μm, and the mean size of the GSNSs was 80.31 μm, which is consistent with the technical data of the GSNSs provided by the supplier.

### 2.2. Specimen Preparation

Based on previous findings provided in literature [34,35], efforts were made to carefully design the mix proportions of siliceous sacrificial materials, as shown in Table 2. The addition of GSNSs was applied in this experiment to further improve the mixtures of the tested materials. The mixtures of the tested cement paste were labelled as SP0, SP1, SP2, and SP3 for convenience, while the mixtures of the tested mortar were labelled into SM0, SM1, SM2, and SM3, to demonstrate the corresponding addition of GSNSs as 0 wt.%, 0.03 wt.%, 0.1 wt.% and 0.3 wt.%. Weight of binders (cement and supplementary cementitious materials) was used to calculate the content of GSNSs in the mixtures. It should be pointed out that various amounts of superplasticizer were added to the mixtures of the tested cement paste and mortar, in order to ensure a consistent workability of the different mixtures, and the slump flow was maintained at approximately 240 mm for each mixture of the tested cement paste and mortar.

The prismatic specimens (size: 40 × 40 × 160 mm^3^) were thereby cast based on the above mixtures. Plastic sheets were used to cover the moulds after casting. Then, the moulds were cured under an ambient environment for 24 h. A relative humidity of above 95% and a temperature of 21 ± 1 °C were prepared for the standard curing room, according to the Chinese standard GB/T 50081-2002 [36]. After removing the moulds, the specimens were cured over 28 days within the curing room. For each mixture, 20 prismatic specimens were produced.

### 2.3. Testing Methods

According to GB/T 17671-1999 [37], compressive strength and flexural strength of the tested materials that contain various amounts of GSNSs were identified to explore the effect of GSNSs on siliceous sacrificial materials. The pore size distribution and porosity of the tested materials that contain various amounts of GSNSs was determined using a mercury intrusion porosimetry (MIP) of Micromeritics AutoPore IV 9510, for the purpose of undertaking a quantitative analysis of the relation between siliceous sacrificial mortar and GSNSs. During elevated temperature exposure, a simultaneous thermal analyser (NETZSCH STA449 F3, NETZSCH, Selb, Germany) was used for differential scanning calorimetry (DSC) and thermogravimetric analysis (TGA) of the tested materials that contain various amounts of GSNSs, in order to estimate the enthalpy evolution and mass of siliceous sacrificial materials. The experimental conditions were, at the 10 °C/min to 1300 °C heating rate, in nitrogen circumstance, and at standard atmospheric pressure. An agate mortar was used to grind the specimens for TGA tests into powder by hand.

On the basis of heat transfer and the Equation $\upsilon =\dot{Q}/\left(\rho \cdot A\cdot \Delta H\right)$, υ is the ablation velocity of the tested materials, $\dot{Q}$ is the heat flux; ρ is the density; A is the ablating area; ΔH is the decomposition enthalpy. The DSC curve was integrated to estimate the enthalpy of siliceous sacrificial materials [38]. In this experiment, 25 °C was set was the zero point of enthalpy. The decomposition temperature could be estimated via a radiant electrical furnace (Nanjing Yayue Hardware and Machine Co. Ltd., Nanjing, China), which was controlled by computer, at a heating rate of 5 °C/min. Subsequent effort can be made to determine the decomposition enthalpy. In this way, the ablation velocity of the tested materials can be analysed on a qualitative basis.

It is worth pointing out that measurements were performed repeatedly for three times on DSC experiments, TGA, flexural strength and pore structure, for the purpose of improving the accuracy of the results. In addition, measurements were carried out repeatedly for six times to measure the compressive strength. Only the average values were reported herein.

### 2.4. Molecular Dynamics Simulation

#### 2.4.1. Construction of the Model

**Figure 4:**
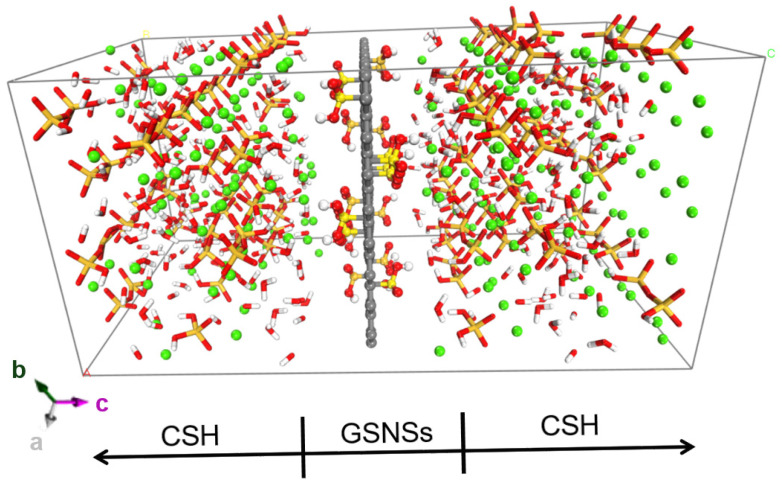
Molecular dynamics model of CSH/GSNSs composition, with lattice parameters a = 21.6 Å, b = 22.52 Å, c = 44 Å, and α = β = γ = 90° and periodic boundaries implemented in x, y, and z direction.

With hydrophilic functional groups, the GSNSs can act as the hydration template during cement hydration, resulting in the packing of cement hydrates around GSNSs. That can be illustrated in Figure 4, the model of CSH/GSNS’s composition.

The pristine graphite unit cell with lattice parameters a = 2.46 Å, b = 4.26 Å, c = 3.4 Å, and α = β = γ = 90° was replicated eight times along a-axis and five times along b-axis to construct the graphitic sheet. Then the graphitic sheet was functionalized by sulfonated groups (–SO_3_H) to reach the oxidization rate of 5%.

The analogue of CSH gel, Tobermorite11Å mineral, was used to be the starting structure for the construction of the CSH model. The detailed processed procedure is available in Ref. [39]. In this work, the chemical formula of the CSH gel obtained was (CaO)_1.65_·(SiO_2_) 1.66H_2_O, matching well with the results obtained by SANS test [40], (CaO)_1.7_(SiO_2_)·1.8H_2_O. Polymerization degree (Q_1_ = 67.2%, Q_2_ = 31.5% and Q_0_ = 1.3%) and mean chain length (MCL = 2(Q_2_/Q_1_ + 1)) of 2.94 fall in the range of the experimental results of Nuclear Magnetic Resonance (NMR) [41,42,43,44,45].

#### 2.4.2. Computation Procedure

Reactive force field was applied to simulate the structure evolution, chemical reaction, and dynamics of GSNSs and CSH gel at 1500 K. The breakage and formation of chemical bonds were determined by bond order scheme, during which the potential parameters were updated continuously according to interatomic distance to achieve the simulation of chemical reactions. Reactive force field includes diagonal terms to describe energy of the deformation of interatomic bond, bond angle, and torsion angle, and non-bonded terms related to Coulombic interactions and van der Waal. It has been found to be applicable to CSH and graphitic structure [23,46,47,48].

## 3. Results and Discussion

### 3.1. Mechanical Strength

#### 3.1.1. Flexural Strength

**Figure 5:**
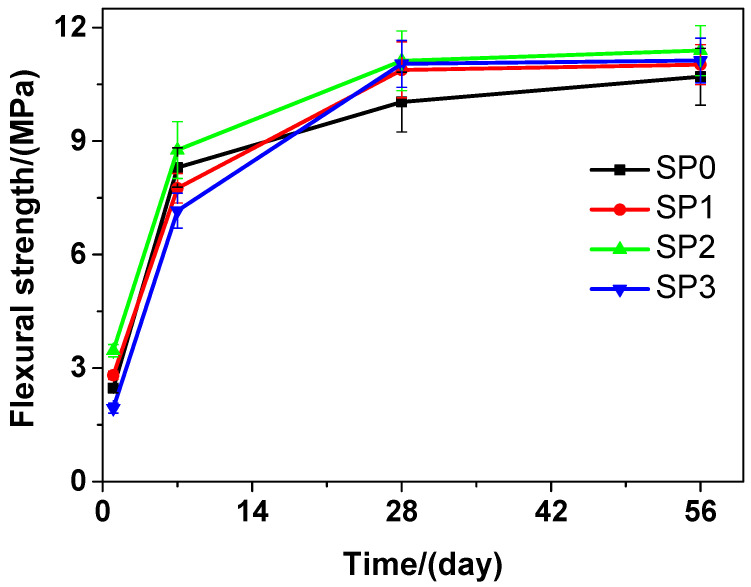
The flexural strength of siliceous sacrificial cement paste at different curing time.

Figure 5 presents that at various curing time, the flexural strength of the tested cement paste that contains various amounts of GSNSs is different. It can be seen that as the curing time increased, the flexural strength was also on the rise. With the passage of the time, the flexural strength of SP2 was always the highest compared to that of SP0, SP1, and SP3. At the curing time of 1 day, in terms of the flexural strength, SP1 and SP2 performed better than SP0, but SP0 performed better than SP3. In terms of the flexural strength, at the 7-day curing time, SP0 performed better than SP1 and SP3, while SP2 performed better than SP0. At the 28-day and 56-day curing time, SP1, SP2, and SP3 performed better than SP0 in terms of the flexural strength. At the 1-day, 7-day, 28-day and 56-day curing time, the flexural strength of SP2 was 40.08%, 5.54%, 9.80%, and 6.45% respectively, all higher than that of SP0, just owing to the addition of 0.1 wt.% GSNSs. SP2 always performed the best at different curing time in terms of the flexural strength, which suggested that 0.1 wt.% GSNSs brought the optimal flexural strength in the tested cement paste. At the 28-day curing time, the flexural strengths of SP0, SP1, SP2, and SP3 were 10.03, 10.88, 11.12, and 11.04 MPa, respectively, suggesting an increase of 8.47%, 10.87%, and 10.07% in the flexural strength of the tested cement paste, when the contents of GSNSs were 0.03 wt.%, 0.1 wt.% and 0.3 wt.%, respectively. At the curing 28-day time, the flexural strength of the tested cement paste was higher than that of its ferro-siliceous counterpart [49]. In addition, SP1 and SP3 maintained at almost the same level in terms of their flexural strength during various curing time points.

**Figure 6:**
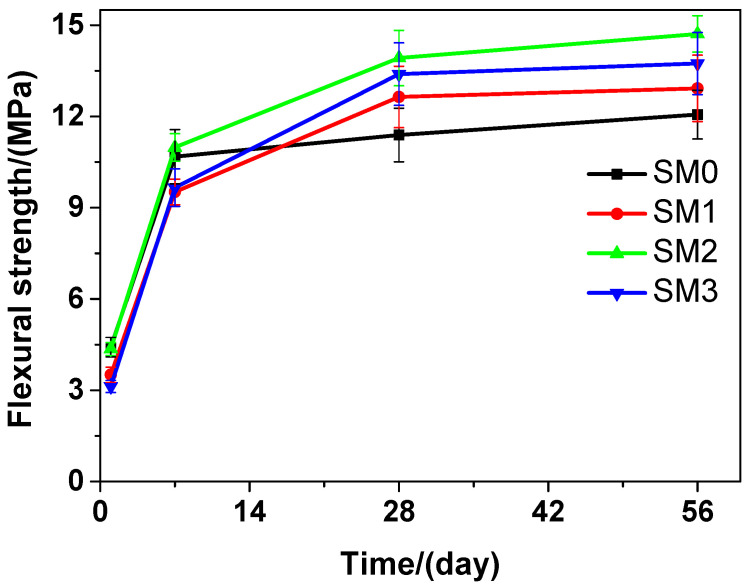
The flexural strength of siliceous sacrificial mortar at different curing time.

At various curing time points, obvious changes could be observed in the flexural strength of the tested mortar that contains different amounts of GSNSs, as illustrated in Figure 6. Similar to the tested cement paste, as the curing time proceeded, the flexural strength of the tested mortar is also on rise. The flexural strength of SM2 was the highest compared to that of SM0, SM1, and SM3 at the 7-day, 28-day and 56-day curing time, which suggested that the optimal amount of GSNSs in siliceous sacrificial mortar was also 0.1 wt.% in terms of the flexural strength. At the1-day curing time, SM0 performed better than SM2 in terms of the flexural strength. However, at the 7-day, 28-day and 56-day curing time, the latter was higher than the former. At the 1-day and 7-day curing time, SM2 performed better than SM1 and SM3 in terms of the flexural strength. Nevertheless, at the 28-day and 56-day curing time, the latter was higher than the former. The flexural strength of SM2 was the highest at the 1-day, 28-day and 56-day curing time, indicating that the flexural strength of SM2 was 2.81%, 22.21%, and 16.92% respectively, higher than that of SM at the 7-day, 28-day and 56-day curing time, just owing to the addition of 0.1 wt.% GSNSs. The flexural strengths of SM0, SM1, SM2, and SM3 at the 28-day curing time were 11.39, 12.64, 13.92, and 13.39 MPa, respectively, suggesting an increase of 10.97%, 22.21%, and 17.56% in the flexural strength of the tested mortar, when the contents of GSNSs were 0.03 wt.%, 0.1 wt.% and 0.3 wt.%, respectively. At the 28-day curing time, the flexural strength of the tested mortar was higher than that of its ferro-siliceous counterpart [49].

Therefore, the decrease in the flexural strength of the tested materials at the beginning of curing (1 and 7 days) may be caused by the incorporation of GSNSs. When 0.1 wt.% GSNSs were added into the tested materials at 28-days curing time, an obvious increase of 10.87% (SP2) and 22.21% (SM2), respectively, was thereby caused in the flexural strength of the tested materials. These findings are consistent with the results of Hou et al. [23], Ranjbar et al. [24], and Lv et al. [25]. In terms of flexural strength, the optimal amount of GSNSs in siliceous sacrificial materials was 0.1 wt.%. The flexural strength of cement-based materials is mainly determined by the mechanical performance of matrix and the degree of cement hydration [50]. The addition of GSNSs can improve the degree of tricalcium silicate hydration [31], and thus the degree of cement hydration can be enhanced by GSNSs. Furthermore, the microstructure of sacrificial concrete could be reinforced with the help of GSNSs [51]. At the same time, the incorporation of GSNSs can also enhance the microstructure of the tested materials. Consequently, the incorporation of GSNSs could improve the flexural strength of the tested materials. It should be pointed out the flexural strengths of SP2 and SP3 were nearly the same, while they had different contents of GSNSs. If the content of GSNSs is higher than the optimal amount, they are apt to agglomerate due to their large specific surface area and the strong intermolecular forces, and thus the dispersion of GSNSs becomes poor. Therefore, the flexural strength of the tested cement paste cannot be improved by adding more GSNSs.

#### 3.1.2. Compressive Strength

**Figure 7:**
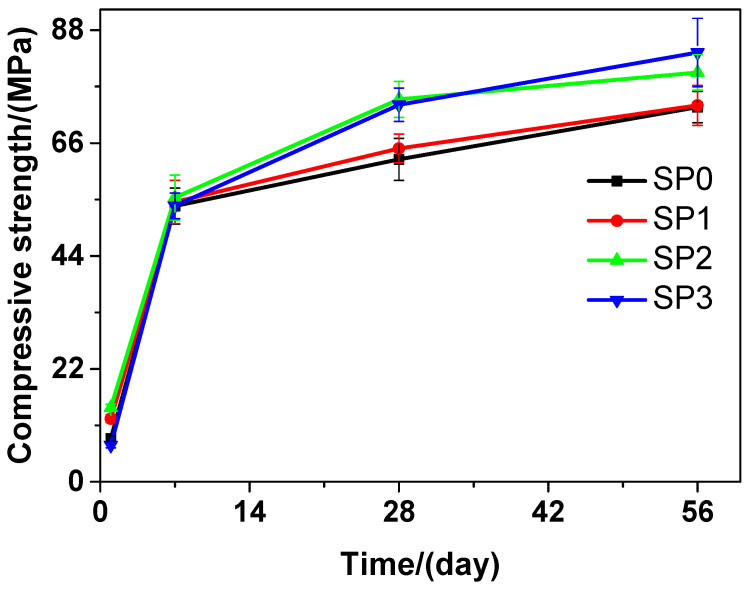
The compressive strength of siliceous sacrificial cement paste at different curing time.

At different curing time points, obvious changes were found in the compressive strength of the tested cement paste that contains different amounts of GSNSs. As shown in Figure 7, as the curing time proceeded, the compressive strength of the tested cement paste also improved. At the 1-day curing time, SP0 performed better than SP3 in terms of the compressive strength, but SP1 and SP2 performed better than SP0 in this aspect. At the 7-day, 28-day and 56-day curing time, SP1, SP2, and SP3 performed better than SP0 in terms of the compressive strength. At the 56-day curing time, SP3 performed better than SP0 in terms of the compressive strength, and achieved the highest level of the compressive strength, which was 14.56%. At the 28-day curing time, the compressive strengths of SP0, SP1, SP2, and SP3 were 62.81, 64.93, 74.52, and 73.46 MPa, respectively, suggesting an increase of 3.38%, 18.64%, and 16.96% in the compressive strength of the tested cement paste, when the contents of GSNSs were 0.03 wt.%, 0.1 wt.% and 0.3 wt.%, respectively. At the 28-day curing time, the tested cement paste performed better than its ferro-siliceous counterpart in terms of the compressive strength [49]. In contrast with SP0, SP1, and SP3, the compressive strength of SP2 was the highest at the 7-day and 28-day curing time, suggesting that the optimal amount of GSNSs in siliceous sacrificial mortar was 0.1 wt.% in terms of compressive strength.

**Figure 8:**
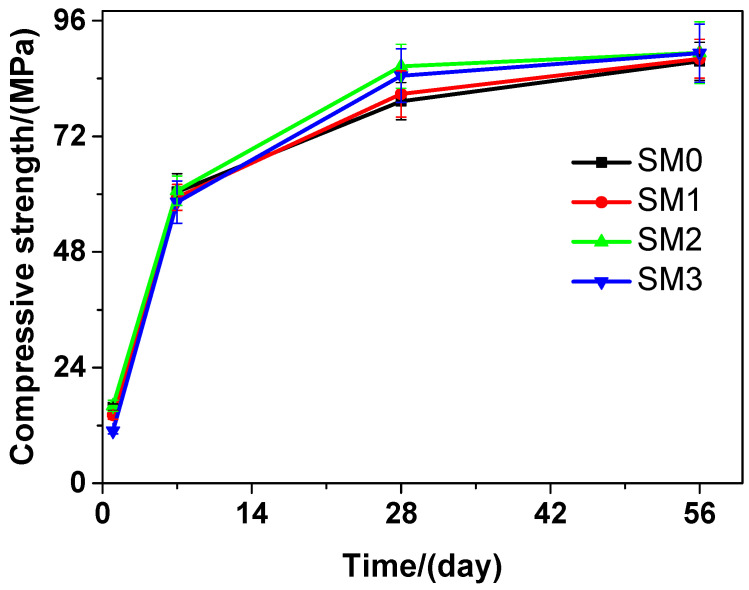
The compressive strength of siliceous sacrificial mortar at different curing time.

Similar to the tested cement paste, as illustrated in Figure 8, the tested mortar also had increase in the compressive strength as the curing time increased. At various curing time points, SM2 always performed better than SM0 in terms of the compressive strength. At the 1-day and 7-day curing time, SM0 performed better than SM1 and SM3 in terms of the compressive strength. However, at the 28-day and 56-day curing time, the latter was higher than the former. At the 28-day curing time, the compressive strengths of SM0, SM1, SM2, and SM3 were 79.28, 80.80, 86.54, and 84.59 MPa, respectively, suggesting an increase of 1.92%, 9.16%, and 6.70% in the compressive strength of the tested mortar, when the contents of GSNSs were 0.03 wt.%, 0.1 wt.% and 0.3 wt.%, respectively. At the 28-day curing time, the tested mortar performed better than its ferro-siliceous counterpart in terms of the compressive strength [49]. Compared to the compressive strength of SM0, SM1, and SM3, the compressive strength of SM2 was always the highest at different curing time, which was 1.90%, 0.43%, 9.16%, and 2.04% higher than that of SM0 at 1, 7, 28, and 56 days, respectively, due to the addition of 0.1 wt.% GSNSs. Therefore, when it comes to compressive strength, the optimal amount of GSNSs in siliceous sacrificial mortar was also 0.1 wt.%. It should be pointed that the compressive strength of SP2 and SP3 was significantly higher than that of SP0 and SP1 at 56-day curing time, but the compressive strength of SM2 and SM3 was a little higher than that of SM0 and SM1 at the same curing time. This was possibly because the improved effects of GSNSs for the tested cement paste were higher than that of the tested mortar.

Thus, the addition of GSNSs at early curing time (1 and 7 days) resulted in the decrease in the compressive strength of the tested materials, while the addition of 0.1 wt.% GSNSs respectively brought an increase of 18.64% (SP2) and 9.16% (SM2) in the compressive strength of the tested materials. This result is consistent with the findings presented in [24] and [25]. In terms of compressive strength, the optimal amount of GSNSs in the tested materials was found to be 0.1 wt.%. GSNSs can improve the degree of tricalcium silicate hydration [31], and hence the addition of GSNSs can also improve the degree of cement hydration. The microstructure of sacrificial concrete can be reinforced and toughened by GSNSs [51], and then the microstructure of the tested materials could also be improved with the help of the inclusion of GSNSs. Accordingly, it can be derived that the incorporation of GSNSs has enhancing effects on the compressive strength of the tested materials.

In summary, GSNSs led to improvement in the mechanical strength of the tested materials and 0.1 wt.% GSNSs was discovered to be the optimal amount that can be added in siliceous sacrificial materials. To be specific, the addition of 0.1 wt.% GSNSs resulted in an increase of 9.80% (SP2) and 22.21% (SM2), respectively, in the flexural strength of the tested materials and an increase of 18.64% (SP2) and 9.16% (SM2), respectively, in the compressive strength of the tested materials.

In addition, the bulk density of the investigated sacrificial materials was also conducted in the work. At the 28-day curing time, the bulk density of SP0, SP1, SP2, and SP3 was 2.068, 2.021, 2.093, and 2.082 g/cm^3^, respectively, which suggested that the bulk density of siliceous sacrificial cement paste was nearly the same. The bulk density of SM0, SM1, SM2, and SM3 at the 28-day curing time was 2.191, 2.294, 2.202, and 2.263 g/cm^3^, respectively, which indicated that the bulk density of siliceous sacrificial mortar was also basically the same. That was because they had the nearly same mixtures (see Table 2). Clearly, the bulk density of siliceous sacrificial mortar was higher than that of siliceous sacrificial cement paste.

### 3.2. Pore Structure

#### 3.2.1. Porosity

**Figure 9:**
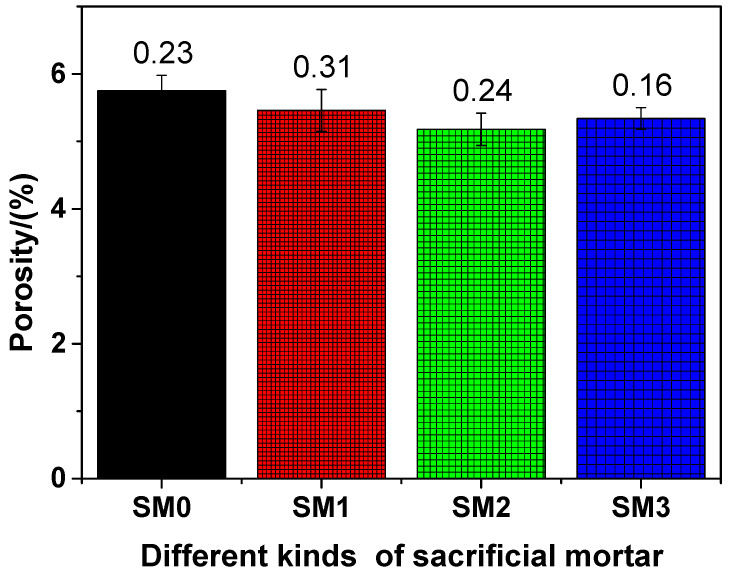
The porosity of different kinds of siliceous sacrificial mortar at curing time of 28 days.

The porosity of different kinds of siliceous sacrificial mortar at the 28-day curing time is shown in Figure 9. As illustrated in Figure 9, as the amount of GSNSs was added, the porosity of the tested mortar decreased, indicating that the increase of GSNSs brought improvement in the pore structure of the tested mortar. Accordingly, GSNSs can lift up the degree of cement hydration [31]. At the same time, the microstructure of sacrificial concrete can be reinforced and toughened by GSNSs [51]. Therefore, the incorporation of GSNSs could improve the pore structure of siliceous sacrificial mortar. The porosities of SM0, SM1, SM2, and SM3 were 5.75%, 5.46%, 5.18%, and 5.34, respectively, at the 28-day curing time, suggesting the decline of 5.04%, 9.91%, and 7.13% in the porosities of the tested mortar, when the contents of GSNSs were 0.03 wt.%, 0.1 wt.% and 0.3 wt.%, respectively. At the 28-day curing time, the porosity of siliceous sacrificial mortar was lower when compared to its ferro-siliceous counterpart [49]. It also can be seen from Figure 9 that the porosity of SM2 was the lowest, which suggested the optimal amount of GSNSs in siliceous sacrificial mortar was 0.1 wt.% in terms of porosity. Obviously, the porosities of different kinds of siliceous sacrificial mortar were negatively correlated with flexural and compressive strength (mechanical strength). Thus, the decreased porosity of siliceous sacrificial mortar might make great contribution to the improvement effect of the addition of GSNSs on their mechanical properties.

#### 3.2.2. Pore Size Distribution

**Figure 10:**
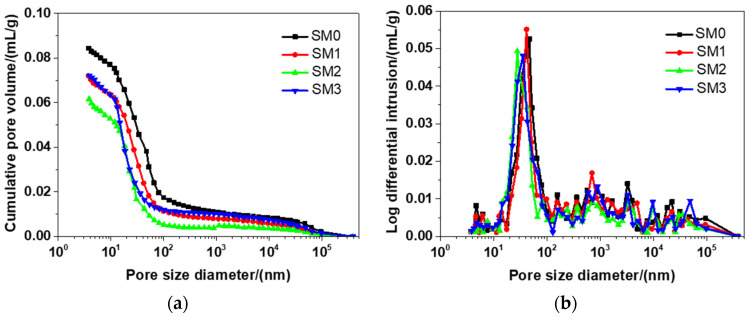
The pore size distribution of different kinds of siliceous sacrificial mortar at curing time of 28 days: (**a**) cumulative pore distribution curve; (**b**) differential pore distribution curve.

Figure 10 presents the distribution of the pore size of different kinds of siliceous sacrificial mortar at the 28-day curing time. As shown in Figure 10a, the tested materials had a similar cumulative pore distribution curve, because they had nearly the same mixtures (see Table 2). There were a number of small peaks in each curve (see Figure 10b), but there was also a typical peak in each curve. The threshold pore diameters of SM0, SM1, SM2, SM3 were 46.91, 41.49, 30.31, and 35.64 nm, respectively, which suggested that the threshold pore diameters of siliceous sacrificial mortar were reduced by 13.06%, 35.39%, and 24.02%, when the contents of GSNSs were 0.03 wt.%, 0.1 wt.% and 0.3 wt.%, respectively. The threshold pore diameter of siliceous sacrificial mortar at the curing time of 28 days was larger when compared to ultra-high-performance concrete with aeolian sand [52]. As the amount of GSNSs added, the threshold pore diameter of the tested mortar reduced, indicating that the addition of GSNSs refined the pore structure of the tested mortar. The reasons why the threshold pore diameter of siliceous sacrificial mortar reduced were the same as the reasons why their porosity decreased, as mentioned above. In addition, the presence of GSNSs might be the cause for the improvement of pore structure since it also has cracking–bridging effects and nano-filler effects [23].

### 3.3. Thermal Analysis

#### 3.3.1. Thermo Gravimetric Analysis (TGA)

**Figure 11:**
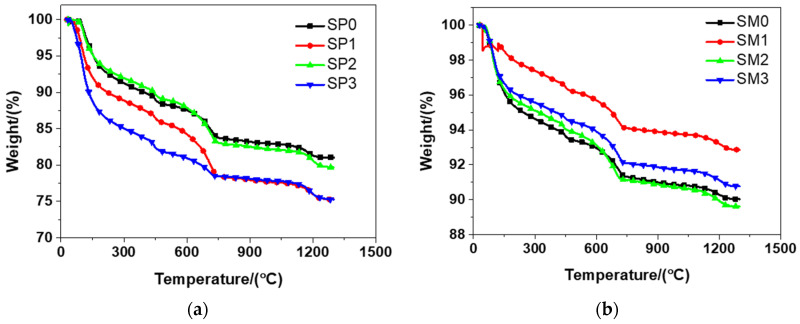
The thermogravimetric analysis of siliceous sacrificial cement paste and mortar: (**a**) siliceous sacrificial cement paste; (**b**) siliceous sacrificial mortar.

According to Figure 11, it can be seen from the results that the tested materials had a similar weight evolution, which could be ascribed to the similar mixtures (see Table 2) of them. Sacrificial cement paste and mortar, no matter ferro-siliceous or siliceous, shared the same changing tendency, as shown in literature [49]. It was observed that there was a rapid weight decline in the TGA for the tested materials in the range of 25–150 °C. At elevated temperatures, self-compacting cement paste shared the similar result [53]. The main cause of this phenomenon was the loss physically bound water and evaporable water in the tested materials. Between 105 and 700 °C, the loss of dehydration products and chemically bound water resulted in the mass loss indicated from TGA [54]. At 700 °C, the weight of the tested materials encountered a significant loss. The decarbonation of calcium carbonate was considered to be the major cause of this phenomenon. The curves of TGA evolved in a smooth manner after that temperature. Because of the melting of Portland cement, the tested materials had another sharp drop in their weight at about 1200 °C. When the temperature arrived at 1300 °C, the total loss of weight in SP0, SP1, SP2, and SP3 was respectively 18.97%, 24.72%, 20.31%, and 24.73%. At the same time, the total weight loss of SM0, SM1, SM2, and SM3 was respectively 9.98%, 7.15%, 10.38%, and 9.23%. Overall, compared to siliceous sacrificial mortar, siliceous sacrificial cement paste had a higher level of weight loss, when the temperature ran up to 1300 °C.

#### 3.3.2. Differential Scanning Calorimetry (DSC)

**Figure 12:**
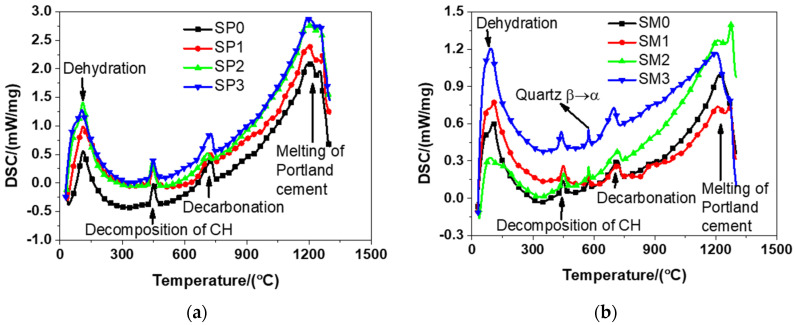
The differential scanning calorimetry results of siliceous sacrificial cement paste and mortar: (**a**) siliceous sacrificial cement paste; (**b**) siliceous sacrificial mortar.

According to Figure 12, it can be seen from the results that there was a similar trend between the DSC patterns of the tested materials, primarily because of the essential similarity between their cement hydration products. The tested materials encountered dehydration at about 100 °C that was featured with the loss physically bound water and evaporable water. The same finding was observed by Bazant and Kaplan [54] in their study. The decomposition of CH occurred in the range from 400 to 600 °C. The same finding was also described in relevant previous studies [54]. The change of crystalline of quartz from β- to α-quartz at about 580 °C was also reported in the study by Chase [38]. At approximately 700 °C, calcium carbonate decarbonized, exactly meeting the conclusion drawn by Bazant and Kaplan [54]. To be specific, they found the occurrence of the decomposition of calcium carbonate from 600 to 900 °C [54]. At about 1200 °C, it was detected that the melting of Portland cement occurred, which was a phenomenon reported in the experiment results of reference [38]. It should be emphasized that there was generally a continual process for the dehydration of cement hydration products in the range of 100–850 °C. In addition, it should be pointed out that the decomposition of GSNSs was not identified in the DSC results of the tested materials. That might be because, (1) the content of GSNSs in the tested materials is too low to identify by DSC analysis; (2) the decomposition temperature of GSNSs is the same as those components of the tested materials, and thus their decomposition curve may be covered; and (3) the decomposition temperature of GSNSs is not in the range of the tested temperature by DSC analysis. Further research is needed to reveal the specific reasons.

### 3.4. Ablation Behaviour

**Figure 13:**
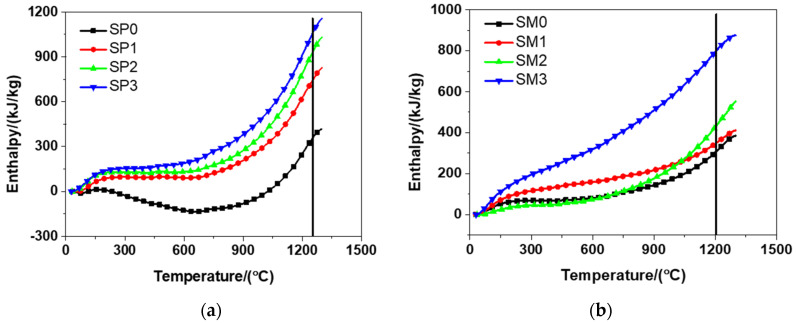
The enthalpy and decomposition enthalpy of siliceous sacrificial cement paste and mortar: (**a**) siliceous sacrificial cement paste; (**b**) siliceous sacrificial mortar.

When using the electrical furnace to carry out high temperature tests, it was found that the decomposition temperatures of the tested materials were about 1250 °C and 1210 °C, respectively. The enthalpy of the tested materials was acquired according to the DSC results illustrated in Figure 12 and is presented in Figure 13. It can be observed from Figure 13 that the inclusion of more GSNSs resulted in the increase in the enthalpy of the tested materials, which was in accordance with the experimental findings of the study on the ferro-siliceous counterpart [49]. The decomposition enthalpy of SP0, SP1, SP2, SP3 was 352.37, 737.56, 924.30, and 1052.30 kJ/kg, respectively, suggesting that the increase of GSNSs was followed by the increase in the decomposition enthalpy of the tested cement paste. Compared to SP0, the decomposition enthalpy of the tested cement paste had a rise of 109.31%, 162.31%, and 198.63%, respectively. The decomposition enthalpy of SM0, SM1, SM2, and SM3 was 314.53, 354.03, 439.91, and 806.64 kJ/kg, respectively, suggesting that the inclusion of more GSNSs was the inducement of the increase in the decomposition enthalpy of the tested mortar. Compared to SM0, the decomposition enthalpy of the tested mortar rose by 12.56%, 39.86%, and 156.46%, respectively.

According to the formula $\upsilon =\dot{Q}/\left(\rho \cdot A\cdot \Delta H\right)$, ablation velocity is inversely proportional to density, ablating area and decomposition enthalpy, but directly proportional to heat flux. With the addition of more GSNSs, the decomposition enthalpy of the tested materials showed an upward trend. Therefore, due to the addition of GSNSs, a decline was found in the ablation velocity of the tested materials. Compared to SP0, the decomposition enthalpy of the tested cement paste rose by 109.31%, 162.31%, and 198.63%, respectively, indicating a decline of 52.22%, 61.88%, and 66.51% in the ablation velocity of the tested cement paste, when the contents of GSNSs were 0.03 wt.%, 0.1 wt.%, and 0.3 wt.%, respectively. Compared to SM0, the decomposition enthalpy of the tested mortar had a rise of 12.56%, 39.86%, and 156.46%, respectively, which suggested a decline of 11.16%, 28.50%, and 61.01% in the ablation velocity of siliceous sacrificial mortar, when the contents of GSNSs were 0.03 wt.%, 0.1 wt.%, and 0.3 wt.%, respectively. Consequently, due to the addition of GSNSs, it was found that there is a significant decline in the ablation velocity of the tested materials. In this regard, it is possible to enhancing the safety of nuclear power plants through extending the melt-through time.

### 3.5. Molecular Dynamics Investigation

**Figure 14:**
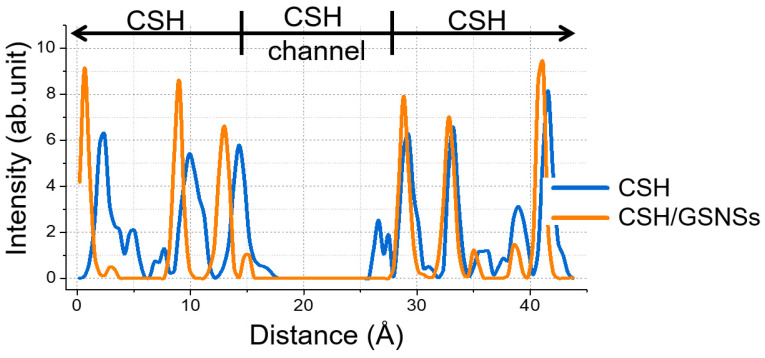
Atomic distribution of silicon along z direction.

Molecular dynamics was performed to probe the mechanism how the GSNSs works in the CSH/ GSNSs composite to increase the resistance to high temperature. Silicon constitutes the skeleton of CSH gel. The atomic distribution of silicon along c-axis is shown in Figure 14, illustrating the deformation and displacement of the CSH. With respect to pristine sample, the peaks were much weaker as compared to the sample with GSNSs, meaning the breakage of layered structure of CSH gel to a greater extent. The diffusion of silicate skeleton into interlayered region implied the melt of the gel at the high temperature.

Calcium and water molecules, with weaker stability than silicate skeleton, are much more likely to diffuse and even escape from the CSH gel at elevated temperature, resulting in the deterioration and damage of CSH gel. Herein, the dynamics of calcium and water molecules is characterized by means of mean square displacement (MSD), as determined by the following Equation.

(1) $$\mathrm{MSD}\left(t\right)=<{\left|r_{i}\left(t\right)-r_{i}\left(0\right)\right|}^{2}>$$

where *r_i_* (*t*) is the position of atom *i* at time *t*.

**Figure 15:**
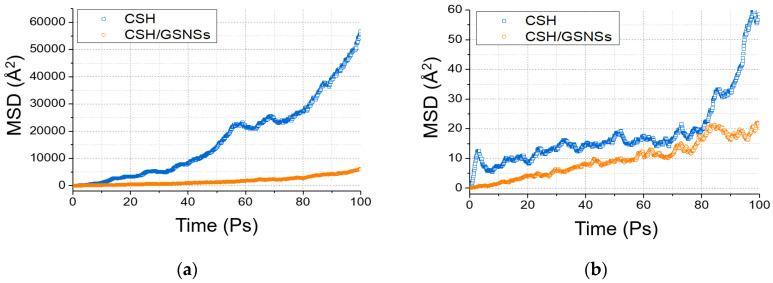
Mean square displacement: (**a**) water, and (**b**) calcium.

As depicted in Figure 15, the MSD profiles increased with the relaxation time, indicating the movement of calcium and water molecules. During the relaxation time of 100 ps, the displacement of water molecules was up to around 23 nm for the pristine sample, resulting in the escape from the structure, while the displacement was around 7 ns for the sample with GSNSs. It indicated that the evaporation rate of water in the hydration system could be reduced by the GSNSs, which would increase phase transition temperature. Furthermore, the addition of GSNSs also restricted the diffusion and escape of calcium, contributing to the stability of the CSH gel.

**Figure 16:**
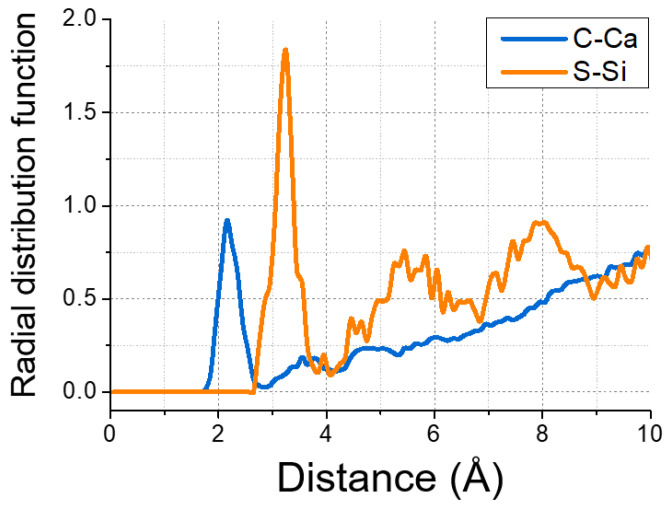
Radial distribution function of C–Ca and S–Si.

The affinity between GSNSs and CSH gel, even at 1500 K, ensured the reinforced effect of GSNSs on the CSH gel system. Figure 16 shows the radial distribution function (RDF) of C–Ca and S–Si to characterize the interatomic spatial correlation, and to estimate the interaction between GSNSs and CSH. Sharp peaks of C–Ca RDF profiles could be observed at the distance of 2.15 Å, implying the packing of calcium on the GSNS’s sheets. This was because that the negatively charged sulfonated groups of GSNSs grabbed the dissociated calcium. Moreover, the GSNSs were interacted with CSH gel by the chemical reaction between sulfonated groups and silicate tetrahedron, as illustrated in S–Si RDF profile, which suggested the formation of S–O–Si bonds.

In summary, it was found that the addition of GSNSs in the tested materials can improve their compressive strength, flexural strength and decomposition enthalpy, and also lower their ablation velocity, threshold pore diameter and porosity. Better pore structure and properties were also found in siliceous sacrificial cement paste and mortar with GSNSs. Considering the mechanical properties, pore structure, and ablation behavior of the tested materials, 0.1 wt.% GSNSs was verified to be the optimal amount. The affinity between GSNSs and CSH gel, even at 1500 K, ensured the reinforced effect of GSNSs on the CSH gel system. These findings are expected to be useful for the development of new types of siliceous sacrificial materials and concrete that contain GSNSs.

## 4. Conclusions

The main focus of this study is put on the effects of GSNSs on properties of siliceous sacrificial materials. Investigation was carried out to examine the influence of various amounts of GSNSs on the mechanical strength, pore structure, and thermal analysis of the tested materials. Molecular dynamics was also used to clarify the mechanism how the GSNSs work in the CSH/GSNSs composite to increase the resistance to high temperature. The following conclusions were drawn based on the experiment results:

(1) The flexural strengths of SP0, SP1, SP2, and SP3 at the 28-day curing time are 10.03, 10.88, 11.12, and 11.04 MPa, respectively, suggesting an increase of 8.47%, 10.87%, and 10.07% in the flexural strength of the tested cement paste, when the contents of GSNSs are 0.03 wt.%, 0.1 wt.% and 0.3 wt.%, respectively.
(2) At the 28-day curing time, the flexural strengths of SM0, SM1, SM2, and SM3 are 11.39, 12.64, 13.92, and 13.39 MPa, respectively, indicating an increase of 10.97%, 22.21%, and 17.56% in the flexural strength of the tested mortar, when the contents of GSNSs are 0.03 wt.%, 0.1 wt.% and 0.3 wt.%, respectively.
(3) The compressive strengths of SP0, SP1, SP2, and SP3 at the 28-day curing time are 62.81, 64.93, 74.52, and 73.46 MPa, respectively, suggesting an increase of 3.38%, 18.64%, and 16.96% in the compressive strength of the tested cement paste, when the contents of GSNSs are 0.03 wt.%, 0.1 wt.% and 0.3 wt.%, respectively.
(4) The compressive strengths of SM0, SM1, SM2, and SM3 at the 28-day curing time are 79.28, 80.80, 86.54, and 84.59 MPa, respectively, suggesting an increase of 1.92%, 9.16%, and 6.70% in the compressive strength of the tested mortar, when the contents of GSNSs are 0.03 wt.%, 0.1 wt.% and 0.3 wt.%, respectively.
(5) The porosities of SM0, SM1, SM2, and SM3 at the 28-days curing time are 5.75%, 5.46%, 5.18%, and 5.34, respectively, suggesting a decline of 5.04%, 9.91%, and 7.13% in the porosities of siliceous sacrificial mortar, when the contents of GSNSs are 0.03 wt.%, 0.1 wt.% and 0.3 wt.%, respectively.
(6) The threshold pore diameters of SM0, SM1, SM2, SM3 are 46.91, 41.49, 30.31, and 35.64 nm, respectively, suggesting a decline of 13.06%, 35.39%, and 24.02% in the threshold pore diameters of siliceous sacrificial mortar, when the contents of GSNSs are 0.03 wt.%, 0.1 wt.% and 0.3 wt.%, respectively.
(7) When the temperature runs up to 1300 °C, the total weight loss of SP0, SP1, SP2, SP3 was 18.97%, 24.72%, 20.31%, and 24.73%, respectively. At the same time, the total weight loss of SM0, SM1, SM2, SM3 was 9.98%, 7.15%, 10.38%, and 9.23%, respectively. Accordingly, compared to the tested mortar, the weight loss of the tested cement paste is higher.
(8) The tested materials have a similar trend in terms of the DSC patterns, because the cement hydration products of them are essentially the same. In general, there is a continual process for the dehydration of cement hydration products in the range of 100–850 °C.
(9) Due to the inclusion of more GSNSs, the decomposition enthalpy of the tested materials is improved. The decomposition enthalpy of SP0, SP1, SP2, SP3 is 352.37, 737.56, 924.30, and 1052.30 kJ/kg, respectively, suggesting an increase of 109.31%, 162.31%, and 198.63% respectively in the decomposition enthalpy of the tested cement paste. The decomposition enthalpy of SM0, SM1, SM2, SM3 is 314.53, 354.03, 439.91, and 806.64kJ/kg, respectively, indicating an increase of 12.56%, 39.86%, and 156.46% respectively in the decomposition enthalpy of the tested mortar.
(10) Due to the inclusion of more GSNSs, the ablation velocity of the tested materials is reduced. The addition of 0.03 wt.%, 0.1 wt.%, and 0.3 wt.% GSNSs results in a decline of 52.22%, 61.88%, and 66.51% respectively in the ablation velocity of the tested cement paste, and a decline of 11.16%, 28.50%, and 61.01%, respectively, in the ablation velocity of siliceous sacrificial mortar.
(11) The optimal amount of GSNSs is 0.1 wt.%, considering the mechanical properties, pore structure, and ablation behaviour of the tested materials.
(12) The GSNSs contribute to the reinforced effect of GSNSs on CSH gel through the grab of dissociated calcium and water molecules, and the chemical reaction with silicate tetrahedron to produce S–O–Si bonds.

## Figures and Tables

**Table 1:** Chemical composition and physical properties of cement and supplementary cementitious materials.

Materials	Cement	Silica Fume	Fly Ash
Chemical composition	(wt.%)		
CaO	64.46	0.81	8.75
SiO_2_	20.19	94.46	48.04
Al_2_O_3_	5.41	0.89	30.17
Fe_2_O_3_	3.11	0.83	4.44
MgO	0.93	0.76	2.60
SO_3_	1.95	0.25	1.23
K_2_O	0.74		1.59
Na_2_O			1.24
Loss on ignition	3.21	2.0	1.94
Physical properties			
Specific gravity	3.15	2.34	2.21
Specific surface (m^2^/kg)	359.48	2.57 × 10^4^	
28 days Compressive strength (MPa)	61.30		

**Table 2:** The mix proportions of siliceous sacrificial cement paste and mortar (g).

Mixture	Cement	Fly Ash	Silica Fume	Silica Sand	Water	Superplasticizer	GSNSs
SP0	331	182	20	0	150	3.5	0
SP1	331	182	20	0	150	3.7	0.160
SP2	331	182	20	0	150	4.1	0.533
SP3	331	182	20	0	150	4.6	1.599
SM0	331	182	20	1122	150	7.8	0
SM1	331	182	20	1122	150	8.0	0.160
SM2	331	182	20	1122	150	8.4	0.533
SM3	331	182	20	1122	150	8.7	1.599
